# Characterization of short-chain fatty acids in patients with ulcerative colitis: a meta-analysis

**DOI:** 10.1186/s12876-022-02191-3

**Published:** 2022-03-10

**Authors:** Hao-Ming Xu, Hai-Lan Zhao, Gong-Jing Guo, Jing Xu, You-Lian Zhou, Hong-Li Huang, Yu-Qiang Nie

**Affiliations:** 1 Department of Gastroenterology and Hepatology, Guangzhou First People’s Hospital, Guangzhou Medical University, No. 1 Panfu Road, Guangzhou, 510180 Guangdong China; 2 Department of Gastroenterology and Hepatology, The Second Affiliated Hospital, School of Medicine, South China University of Technology, Guangzhou, 510180 Guangdong China; 3 Department of Gastroenterology, Longgang District People’s Hospital, Shenzhen, 518172 Guangdong China

**Keywords:** Short-chain fatty acids, Ulcerative colitis, Meta-analysis

## Abstract

**Background:**

Studies investigating the changes in short-chain fatty acids (SCFAs) in patients with ulcerative colitis (UC) have yielded inconsistent results. We performed a meta-analysis of studies that investigated the alterations in different SCFAs among UC patients to assess their role in the development of UC.

**Methods:**

Three databases were searched for relevant studies published as of April 2021. Results are presented as standardized mean difference (SMD) with 95% confidence interval (95% CI).

**Results:**

Eleven studies were included in the meta-analysis. Compared to healthy subjects, UC patients had significantly lower concentrations of total SCFAs (SMD = − 0.88, 95%CI − 1.44, − 0.33; *P* < 0.001), acetate (SMD = − 0.54, 95% CI − 0.91, − 0.17; *P* = 0.004), propionate, (SMD = − 0.37, 95% CI − 0.66, − 0.07; *P* = 0.016), and valerate (SMD = − 0.91, 95% CI − 1.45, − 0.38; *P* < 0.001). On subgroup analysis based on disease status, patients with active UC had reduced concentrations of acetate (SMD = − 1.83, 95% CI − 3.32, − 0.35; *P* = 0.015), propionate (SMD = − 2.51, 95% CI − 4.41, − 0.61; *P* = 0.009), and valerate (SMD = − 0.91, 95% CI − 1.45, − 0.38; *P* < 0.001), while UC patients in remission had similar concentrations with healthy subjects. Patients with active UC had lower butyrate level (SMD = − 2.09, 95% CI − 3.56, − 0.62; *P* = 0.005) while UC patients in remission had higher butyrate level (SMD = 0.71, 95% CI 0.33, 1.10; *P* < 0.001) compared with healthy subjects.

**Conclusion:**

UC patients had significantly decreased concentrations of total SCFAs, acetate, propionate, and valerate compared with healthy subjects. In addition, inconsistent changes of certain special SCFAs were observed in UC patients with different disease status.

## Background

The term chronic inflammatory bowel disease (IBD) is mainly used to refer to Crohn’s disease (CD) and ulcerative colitis (UC). Crohn’s disease may involve any part of the gastrointestinal tract and typically affects all layers of the bowel wall, while UC is confined to rectum and colon and the pathological changes are typically limited to the mucosal layer [1]. The estimated incidence of CD and UC varies from 26 to 199 cases and 37 to 246 cases per 100,000 people, respectively [1]. The incidence of both diseases has shown an increasing trend in developing countries [2]. Though the etiology remains unclear, both genetic and environmental factors have been implicated in the pathogenesis of IBD. In addition, the dynamic balance between commensal microbiota as well as host defensive responses is believed to play an essential role in the pathogenesis of chronic IBD [3]. Previous studies have found altered microbiota in IBD patients compared to healthy subjects [4–6]. In addition, the disordered cellular metabolism in UC patients, including the oxidation of butyrate and the fermentation of short chain fatty acids (SCFA), showed a strong correlation with alteration of gut microbiota [7, 8].

Represented by acetate, propionate, and butyrate, SCFAs are mainly produced by intestinal microbial fermentation of undigested dietary carbohydrates, especially resistant starches and dietary fiber, and sometimes by dietary and endogenous proteins [9]. SCFAs are important not only for the normal intestinal biology [10] but also for the absorption of sodium and fluid in the colon and the proliferation of colonocytes [11]. Therefore, monitoring the changes in SCFAs concentration may be helpful to understand the relationship between impaired intestinal ecology and UC.

Considering that the SCFAs have an important impact on IBD, we performed a meta-analysis of published studies that investigated the alterations in SCFAs levels in UC patients.

## Material and methods

### Literature search and selection criteria

This meta-analysis was performed in accordance with Preferred Reporting Items for Systematic Reviews and Meta-Analyses (PRISMA) guidelines [12]. PubMed, Embase, and Web of Science databases were systematically searched for studies published as of April 2021, using the following keywords: “inflammatory bowel disease”, “IBD”, “ulcerative colitis”, “UC”, “short chain fatty acid”, “SCFA”, “acetate”, “acetic acid”, “propionate”, “propionic acid”, “butyrate”, “butyric acid”, “valerate”, “valeric acid”, “lactate”, “lactic acid”, “metabolite”, and “metabolism”. No limitations were placed on the language of publication. The reference lists of related studies were manually searched to identify additional studies.

The inclusion criteria were: (1) study design: randomized controlled trials (RCT), cohort studies, case–control studies, or comparative studies; (2) study population: patients diagnosed with UC; (3) outcome measurement: SCFAs. The exclusion criteria were: (1) lack of cross-sectional comparison or longitudinal evaluation of SCFAs concentration; (2) single-arm studies, case series, animal experiments, and literature reviews; (3) Literature with incomplete or unusable data.

### Data extraction

The following information was summarized in a pre-formatted spreadsheet: study design, author names, publication year, sample size, study setting, age, ethnicity, sex, diagnostic criteria for UC, disease extent, and the data of outcome measurements. The authors were contacted in case of any missing information.

### Quality assessment

The overall quality of evidence in the included studies was assessed using the modified Newcastle–Ottawa Scale (NOS) [13]. The scale assesses the following three aspects: patient selection, comparability of the intervention/control group, and outcome assessment [13]. The highest NOS score is 9 points [13]. Studies with more > 5 points are regarded as high-quality studies [13].

### Statistical analysis

Although the indicators reported in each study are the same, the detection methods and the units used for the measurement of SCFAs concentration were different among different studies. Therefore, the fecal SCFAs content in this meta-analysis was counted by absolute value. In order to eliminate the influence of absolute value and the difference of measurement units between studies, the effect size was calculated using standardized mean difference (SMD) with 95% confidence intervals (CIs). Heterogeneity among the included studies was assessed using the Cochran Q statistic and *I*^2^ statistic; *P* value < 0.1 or *I*^2^ > 50% was considered indicative of significant heterogeneity [14]. A random [15] or fixed-effects model [16] was applied to calculate the pooled estimate depending on the heterogeneity among the included studies. Sensitivity analysis was performed to test the potential sources of heterogeneity. Subgroup analysis was performed based on the disease status of UC. The data extracted from the studies included the active and/or remission status of UC (The specific diagnostic and activity criteria are listed in Table 1). Begg’s [17] and Egger’s test [18] were used to assess potential publication bias. Two-tailed *P* value < 0.05 were considered significant. STATA version 12.0 (Stata Corporation, College Station, TX, USA) was used for all analyses.

**Table 1:** Baseline characteristics of patients in the trials included in the meta-analysis

Study	Country	Study design	Case/control (n)	Diagnostic criteria	Disease activity criteria	Disease extent	Method	NOS score
Takaishi H [4]	Japan	Case–control	39/10	Clinical symptoms, laboratory data, and histology	At least 5 loose stools including obvious blood and visible inflammation at endoscopy	Proctitis, left-sided colitis, toal colitis	HPLC	6
James SL [19]	Australia	Cohort	14/10	Standard criteria (No detail)	CAI > 5 [36]	Proctitis, distal colitis, extensive colitis	HPLC	6
Machiels K [20]	Belgium	Case–control	127/87	NA	Partial Mayo Score ≥ 2	Proctitis, left-sided colitis, pancolitis	GC/MS	7
Roediger WEW [21]	UK	Case–control	64/16	Endoscopy and histology	Mild/moderate/severe activity in the mucosa or inflammatory change on biopsy [37]	NA	GC	6
Kumari R [22]	India	Case–control	11/10	NA	Clinical colitis activity index > 3 [38]	Proctitis, left-sided colitis, pancolitis	GC/FID	6
Marchesi JR [23]	Ireland	Case–control	10/13	Clinical symptoms, laboratory data, and histopathology	NA	NA	High resolution H NMR spectroscopy	6
Hove H [24]	Denmark	Case–control	42/20	NA	NA	NA	GLC	6
Bjerrum JT [25]	Denmark	Case–control	44/21	Well-established criteria [39]	Partial Mayo Score ≥ 2	Proctitis, proctosigmoiditis, left-sided Colitis, pancolitis	H NMR spectroscopy	6
Vernia P [26]	Italy	Case–control	18/16	Radiographically and/or endoscopy	NA	NA	GLC	6
Vernia P [27]	Italy	Case–control	62/29	Clinical, radiologic, endoscopic, and histologic findings	Criteria of Truelove and Witts [40]	Total colitis, left-sided colitis, proctosigmoiditis	GLC	7
Treem WR [28]	USA	Case–control	17/12	Standard clinical, radiologic, endoscopic, and histologic criteria	Criteria of Truelove and Witts	NA	GLC	6

Abbreviations: CAI, colitis activity index; GC/FID, gas chromatography/flame ionization detection; GC/MS, gas chromatography mass spectrometry; HPLC, high-performance liquid chromatography; GLC: Gas–liquid chromatography; NA, not available

## Results

### Literature search and study selection

**Fig. 1:**
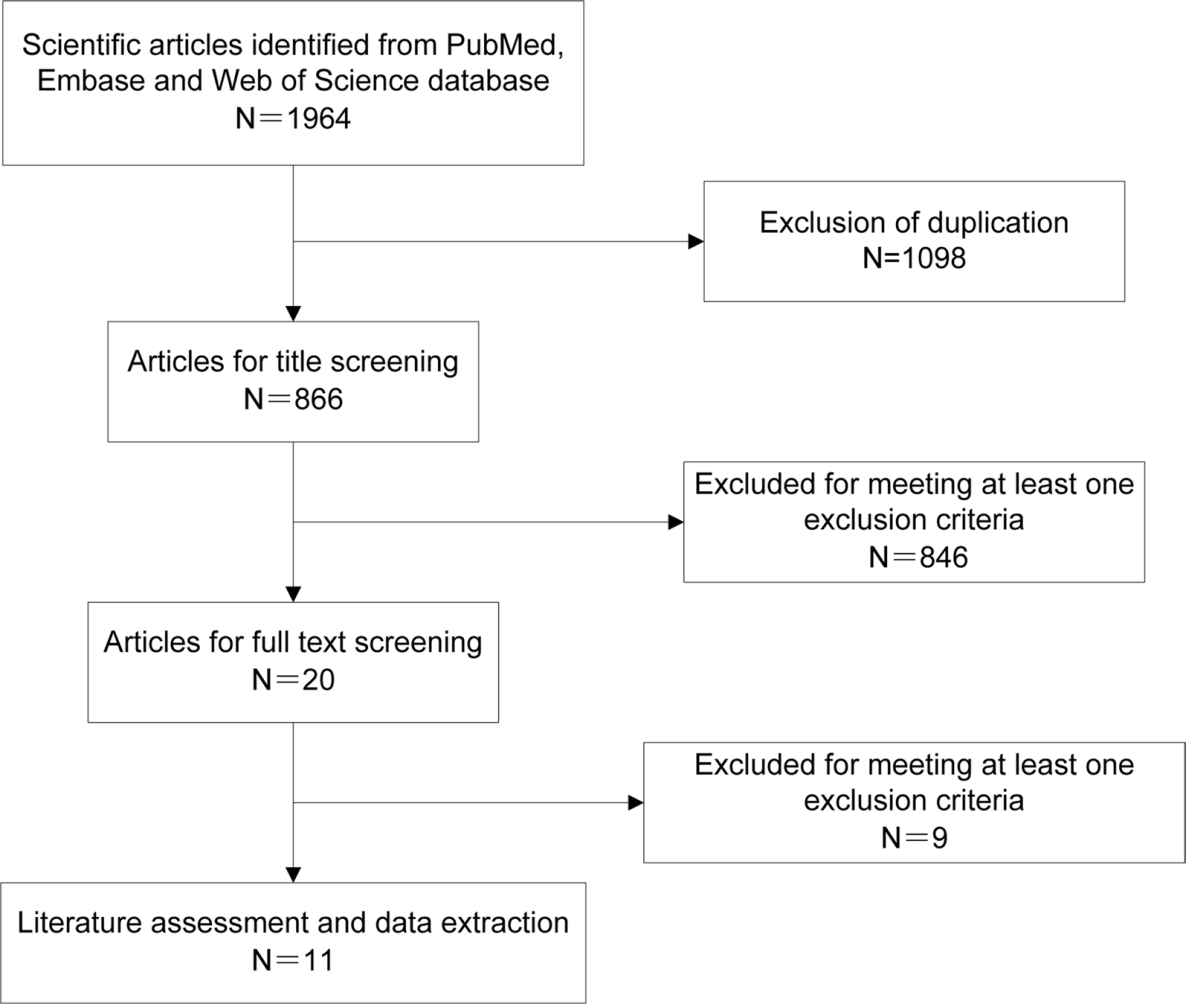
Schematic illustration of the literature search and study-selection criteria

A total of 1964 publications were retrieved on keyword search of the databases. After elimination of duplicate publications, the titles/abstracts of 866 articles were screened. Of these, 846 records were excluded because of various reasons (unrelated to the present study, single-arm study, case series, animal experiments, and literature reviews). Finally, full-text of 20 articles were reviewed, of which 11 studies [4, 19–28] qualified all the inclusion criteria and were included in the meta-analysis (Fig. 1).

### Study characteristics and quality assessment

The year of publication of the selected studies varied from 1982 to 2015. The main characteristics of the included studies are presented in Table 1. The selected studies included one cohort study and ten case–control studies [19]. Eight studies had described the diagnostic criteria of UC, which mainly included: clinical symptoms, laboratory data, endoscopy and histopathology. Eight studies had further analyzed UC patients with active disease and those in remission and the evaluation criteria for disease activity were: Partial MayoScore (n = 2), Clinical colitis activity index (n = 2), Criteria of Truelove and Witts (n = 2), loose stools including obvious blood and overt signs of inflammation at endoscopy (n = 1), mild/moderate/severe activity in the mucosa or inflammatory change on biopsy (n = 1). Six studies had specifically described the lesion sites of UC patients. Gas chromatography (GC) or gas–liquid chromatography (GLC) were the most commonly used methods for analysis of SCFAs in the included studies. All the selected studies had NOS scores of greater than 6, which indicated high quality.

### Total SCFAs

**Fig. 2:**
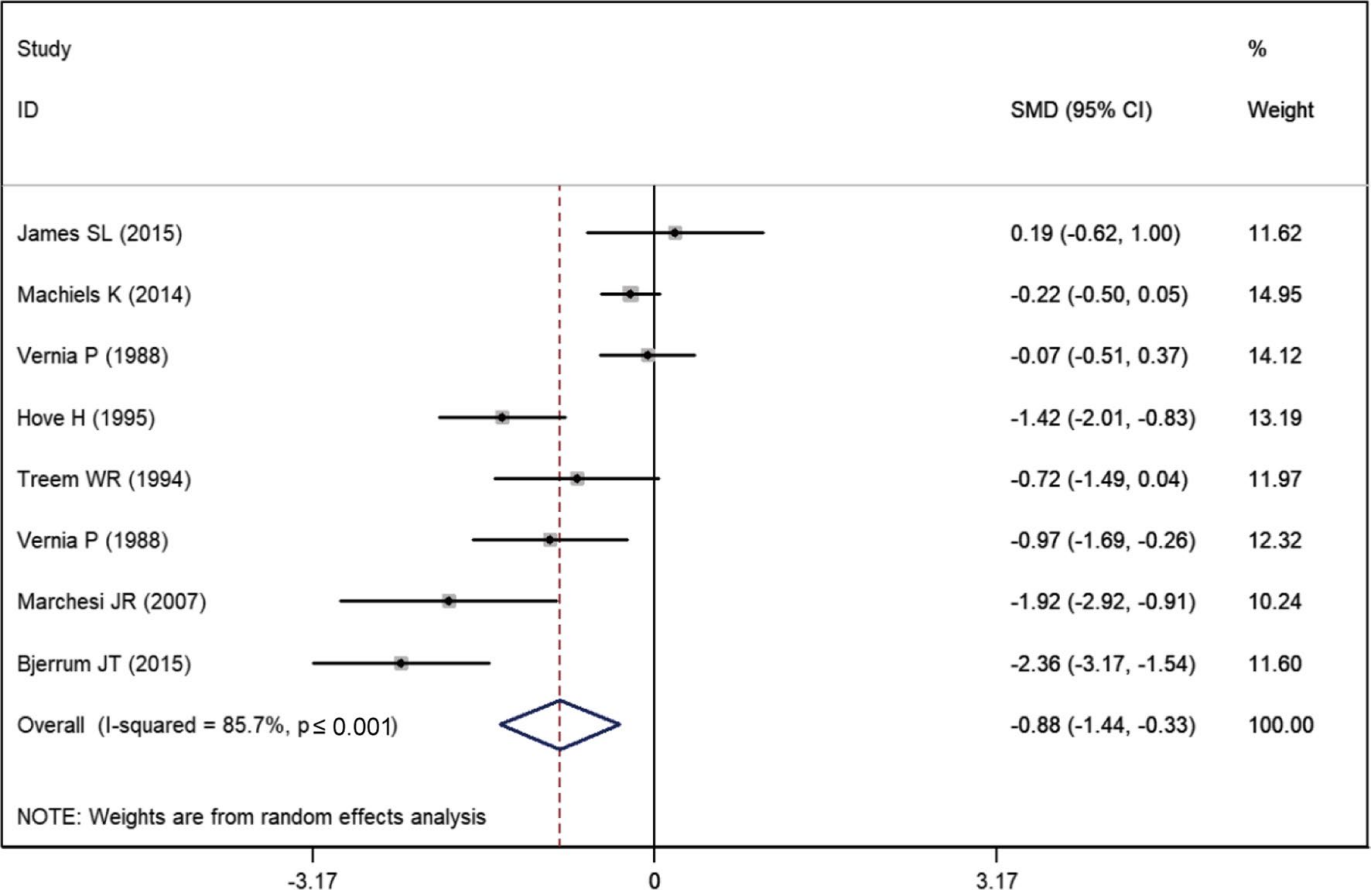
Forest plot showing the comparison of total SCFAs levels between UC patients and healthy subjects

Eight studies reported the data of total SCFAs [19, 20, 23–28]. The mean total SCFAs level in UC and control groups were 67.83 ± 31.11 and 85.60 ± 27.16, respectively. The summarized estimate indicated that the total SCFAs were significantly lower in the UC group when compared to the control group (SMD = − 0.88, 95% CI − 1.44, − 0.33; *P* < 0.001) (Fig. 2). Owing to significant heterogeneity among the included studies (I^2^ = 85.7%, *P* < 0.001), we conducted sensitivity analysis. After exclusion of one study with a small sample size [23], there was a slight change in the overall estimate (SMD = − 0.86, 95% CI − 1.45, − 0.42; *P* < 0.001); however, significant heterogeneity persisted (I^2^ = 81.7%, *P* < 0.001). After exclusion of one study with outliers [26], the combined WMD did not alter substantially (SMD = − 0.86, 95% CI − 1.38, − 0.27; *P* < 0.001); however, the heterogeneity remained significant (I^2^ = 78.5%, *P* < 0.001).

### Acetate

**Fig. 3:**
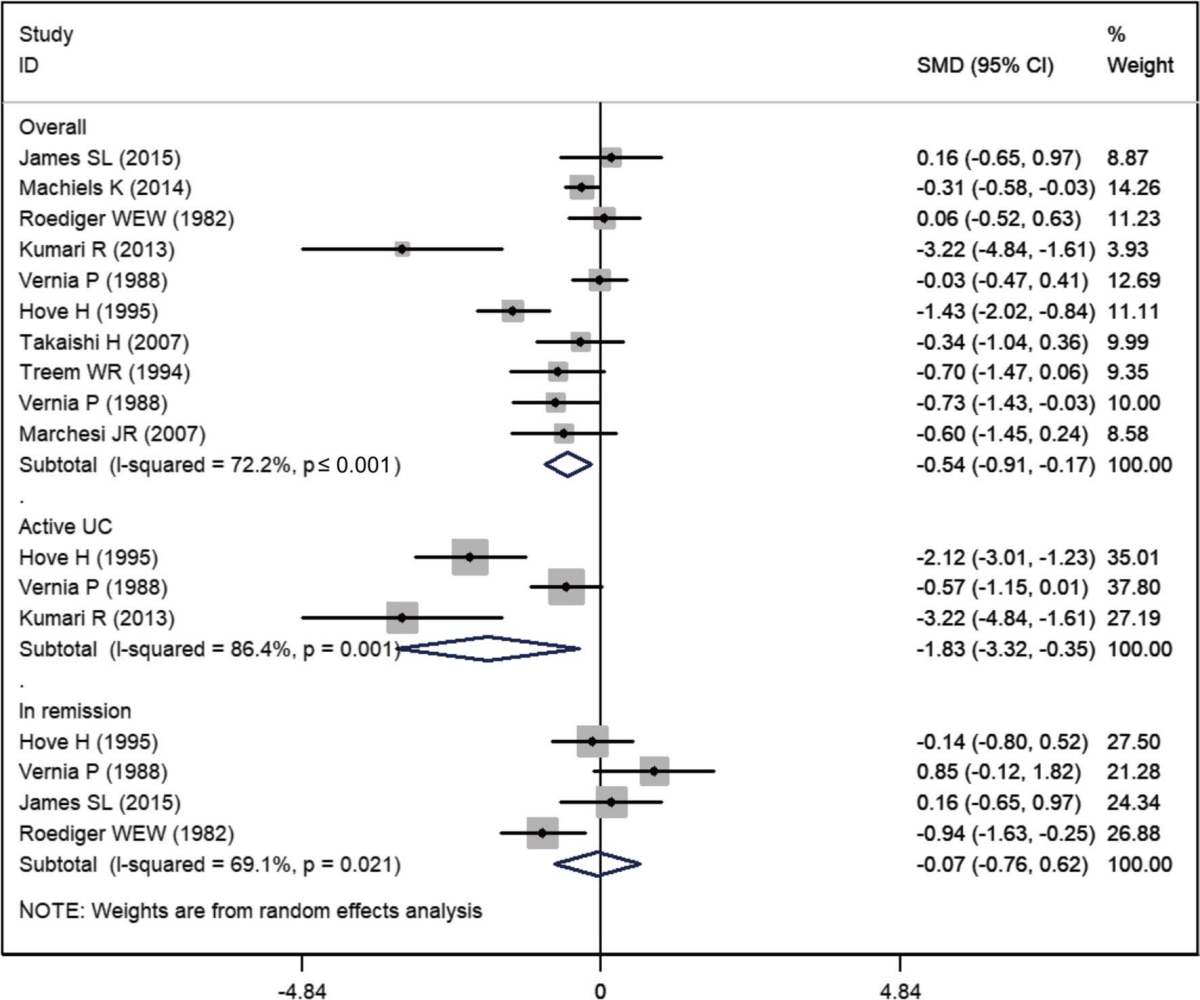
Forest plot showing the comparison of acetate level between UC patients and healthy subjects

Ten included studies reported the data of acetate [4, 19–24, 26–28]. The mean acetate level in UC and control groups were 47.15 ± 23.20 and 56.13 ± 20.23, respectively. Pooled result suggested that the acetate level in the UC group was significantly lower than that in the control group (SMD = − 0.54, 95% CI − 0.91, − 0.17; *P* = 0.004) (Fig. 3). There was significant heterogeneity among the ten studies (I^2^ = 72.2%, *P* < 0.001). On sensitivity analysis, exclusion of trials with small sample size or outliers did not change the result substantially; however, the heterogeneity was still found (data not shown).

We performed subgroup analysis based on the disease status of UC. Reduced acetate level was only seen in patients with active UC (SMD = − 1.38, 95% CI − 3.32, − 0.35; *P* = 0.015) but not in UC patients in remission (SMD = − 0.07, 95% CI − 0.76, 0.62; *P* = 0.841) (Fig. 3).

### Propionate

**Fig. 4:**
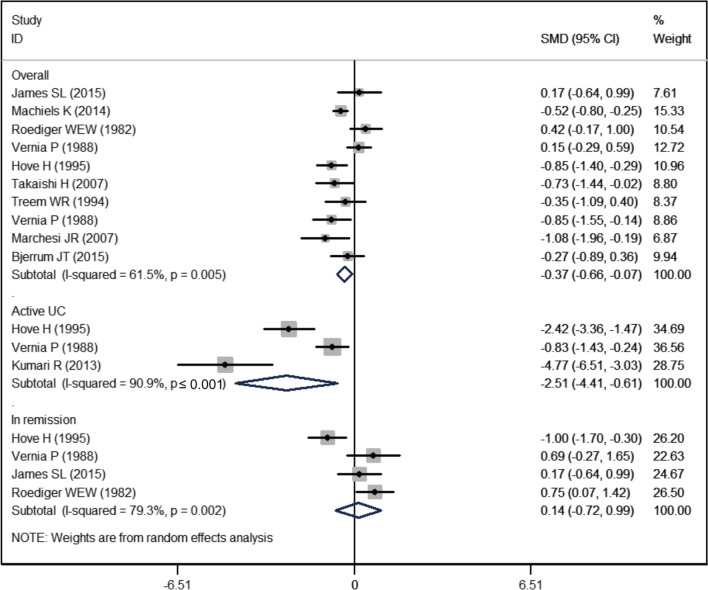
Forest plot showing the comparison of propionate level between UC patients and healthy subjects

Ten studies reported the data of propionate [4, 19–25, 27, 28]. The mean propionate levels in UC and control groups were 15.44 ± 9.57 and 18.12 ± 7.49, respectively. The propionate level in the UC group was significantly lower than that in the control group (SMD = − 0.37, 95% CI − 0.66, − 0.07; *P* = 0.016) (Fig. 4). There was significant heterogeneity among the studies in this respect (I^2^ = 61.5%, *P* = 0.005). However, no valuable information was found in the sensitivity analysis.

On subgroup analysis disaggregated by disease status, the propionate level was significantly reduced in patients with active UC (SMD = − 2.51, 95% CI − 4.41, − 0.61; *P* = 0.009), but not in UC patients in remission (SMD = 0.14, 95% CI − 0.72, 0.99; *P* = 0.756) (Fig. 4).

### Butyrate

**Fig. 5:**
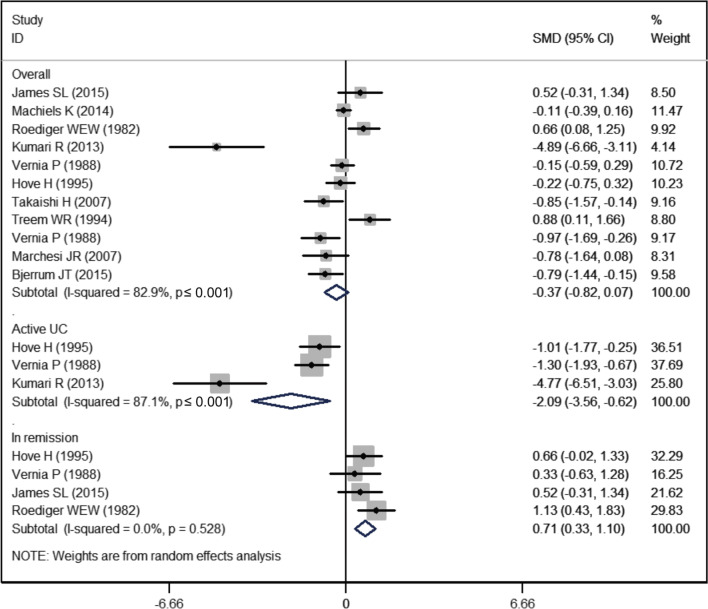
Forest plot showing the comparison of butyrate levels between UC patients and healthy subjects

Ten studies reported the data of butyrate [4, 19–25, 27, 28]. The mean butyrate levels in UC and control group were 11.89 ± 6.62 and 14.41 ± 5.69, respectively. The butyrate level was comparable between the UC group and control group (SMD = − 0.37, 95% CI − 0.82, 0.07; *P* = 0.10) (Fig. 5). There was significant heterogeneity among the studies (I^2^ = 82.9%, *P* < 0.001).

On subgroup analysis disaggregated by disease status, the butyrate level was significantly lower in active UC patients (SMD = − 2.09, 95% CI − 3.56, − 0.62; *P* = 0.005), but higher in UC patients in remission (SMD = 0.71, 95% CI 0.33, 1.10; *P* < 0.001) (Fig. 5).

### Valerate

Six studies had reported data of valerate [19, 23–25, 27, 28]. The mean valerate levels in the UC and control groups were 1.32 ± 1.24 and 2.17 ± 1.21, respectively. The valerate level in the UC group was significantly lower than that in the control group (SMD = − 0.91, 95% CI − 1.45, − 0.38; *P* < 0.001).

### Publication bias

Analysis revealed no significant effect of publication bias on the results of the meta-analysis (Begg’s test: *P* = 0.115; Egger’s test: *P* = 0.327).

## Discussion

In this study, we performed a meta-analysis of data from 11 studies to characterize the alterations in the levels of SCFAs in UC patients. Our results revealed significant decrease in total SCFAs, acetate, propionate and valerate in UC patients compared with healthy subjects. In addition, for certain special SCFAs, inconsistent alterations were found among the UC patients with different disease status. In particular, reduced levels of acetate and propionate were only observed in patients with active UC but not in those in remission. The butyrate level was significantly lower in patients with active UC, but higher in UC patients in remission.

Compared to the healthy population, the consumption of SCFAs, such as acetate and butyrate, was shown to be a distinguishing characteristic of CD patients [23]. SCFAs are usually generated by the fermentation of complex carbohydrates (including fiber, cellulose, and starches) by the intestinal bacteria [23]. The SCFAs, especially butyrate, play a crucial role in the energy supply of intestinal cell wall and stimulate the growth of epidermal cells [29]. Moreover, methylamine and trimethylamine, generated from food degradation in the intestine, have been found to be lower in the watery excreta of CD patients [30]. This indicates the destruction and dysbiosis of the normal bacterial ecology in patients with IBD. The dysbiosis in intestinal bacterial ecology may be attributed to the microbiota disruption caused by T lymphocytes, which are highly reactive to bacteria and promote inflammatory damage of the intestinal brush border [31]. This would impair the protective effect of the inflammatory epithelium barrier, and affect the absorption of nutrients. Compared with healthy subjects, patients with CD or UC show significantly higher amino acid content in the stool due to the malabsorption caused by inflammation.

In the present meta-analysis, we found a significant reduction in the concentrations of total SCFAs, acetate, propionate, and valerate [4, 32]. Peng et al. reported that the level of SCFA is related with an intracellular energy sensor which facilitates the maintenance of the intestinal barrier function [10]. Kumari et al. [22] further confirmed that the fluctuation in butyrate production was strongly associated with the changes in numbers of butyrate producers among UC patients. Shortage of available butyrate may impair the intestinal barrier function, increasing the risk of exposure of the luminal content to the immune system of the host, thereby exacerbating the immune response [10].

Kumari et al. [22] found that the UC fecal samples had significantly decreased producers as well as levels of butyrate and acetate. This demonstrated that the butyrate supply in the colon was impaired, which might result in insufficient energy for colonocytes. The energy deficiency hypothesis of IBD [33] was also supported by the re-emergence of bacteria producing butyrate during the remission phase with a synchronous increase in the concentration of butyrate. Butyrate as well as the bacteria producing butyrate have recently drawn enormous attention in microbiome research. In the present study, total SCFAs, butyrate, acetate, propionate, and valerate levels in UC patients were significantly reduced compared with healthy subjects. Moreover, Machiels et al. [20] also reported reduced concentrations of SCFAs in colonic lumen of patients with UC. Among the bacterial species producing propionate and butyrate, the populations of *Phascolarctobacteria, Roseburia hominis*, and *Faecalibacterium prausnitzii* were shown to be reduced in patients with UC [34, 35]. Considering that the SCFAs or prebiotics can increase the production of SCFAs, alleviate colitis, and protect the function of the intestinal mucosal barrier in UC patients, lack of SCFAs should be a high-risk factor for colitis.

Some limitations of our study should be acknowledged. First, the sample size of some of the included studies was relatively small. Small trials tend to overestimate the effect of an intervention compared to larger trials. Second, there was significant heterogeneity among the studies included in the meta-analysis. This was attributable to the differences in study design, disease status, disease extent, and the methods used for the analysis of SCFAs. Some studies had exclusively considered laboratory findings, clinical findings, or endoscopy findings, which may have led to selection bias regarding the dosage of SCFAs, as patients included in one study may well have different grades of disease severity and extent. Third, the components of SCFAs in UC patients may have been influenced by diet or medications, leading to bias in this meta-analysis.

## Conclusions

In conclusion, the present study suggests that the concentrations of total SCFAs, acetate, propionate, and valerate are significantly reduced in UC patients compared with healthy subjects. In addition, inconsistent alterations of certain special SCFAs were observed among UC patients with different disease status. Due to the limited data, the relationship between the severity or extent of UC lesions and the type and dosage of SCFAs were not investigated in this meta-analysis. Similarly, the potential correlation of biological results and luminal extension with disease prognosis was not analyzed either; further studies focusing on these issues are needed.

## Data Availability

The dataset used and analyzed during the current study is available from the corresponding author on reasonable request.

## Abbreviations

SCFAs: Short-chain fatty acids
UC: Ulcerative colitis
SMD: Standardized mean difference
95% CI: 95% Confidence interval
IBD: Inflammatory bowel disease
CD: Crohn’s disease
NOS: Newcastle–Ottawa Scale
GC: Gas chromatography
